# Supplementary material for: Quantifying hydrogen bonding using electrically tunable nanoconfined water
Source: Nat Commun. 2025 Apr 15;16:3447. doi: 10.1038/s41467-025-58608-6 (PMC12000575; doi:10.1038/s41467-025-58608-6)
Supplement: Supplementary file 1 — Supplementary Information [file 41467_2025_58608_MOESM1_ESM.pdf]

Supplementary Information - Quantifying hydrogen bonding using electrically tunable nanoconfined water

Ziwei Wang[1,2,*], Anupam Bhattacharya[1], Mehmet Yagmurcukardes[3], Vasyl Kravets[1], Pablo Díaz-Núñez[1,2], Ciaran Mullan[1], Ivan Timokhin[1,2], Takashi Taniguchi[4], Kenji Watanabe[4], Alexander N. Grigorenko[1], Francois Peeters[5], Kostya S. Novoselov[1,2,6], Qian Yang[1,2,*], Artem Mishchenko[1,2,*]

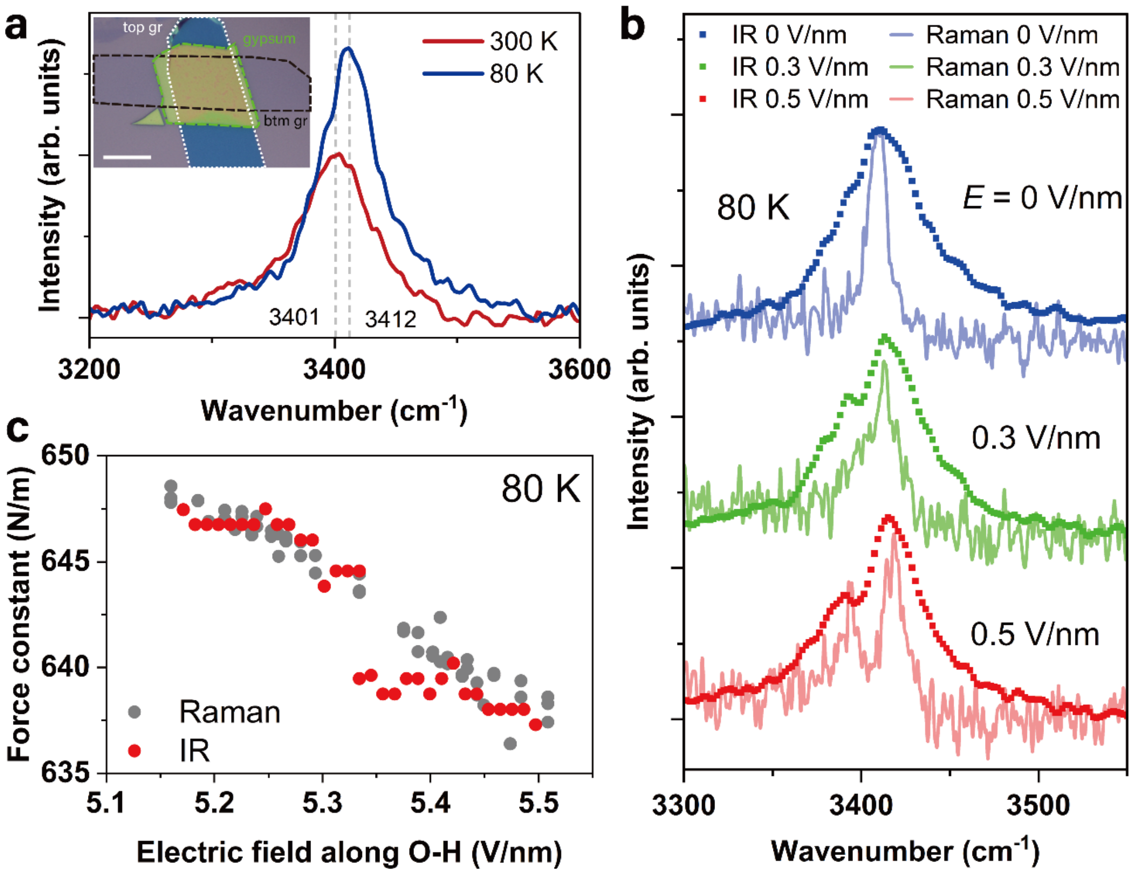

Supplementary Figure 1: Infrared (IR) study on field-induced peak splitting at low temperature. a, Blueshift of water O-H_A stretching peak at 80 K at no external electric field. Inset: optical image of gypsum sample for low-temperature IR measurements. Scale bar 20 µm. **b**, Broadening and splitting of O-H_A stretching peak under external electric field at 80 K (dots). Solid lines are Raman spectra taken from **Fig. 2c** in main text at E_{ext} = 0, 0.3, and 0.5 V/nm, for comparison. **c**, FTIR peak positions for O-H stretching vibration (red). X-axis plots the total electric field along O-H bond, by adding the local O-H_A field E_{HB} = 4.97 V/nm, and taking into account the geometry of H_2O molecules in gypsum. Grey dots correspond to Raman peak positions from **Fig. 3a** in main text.

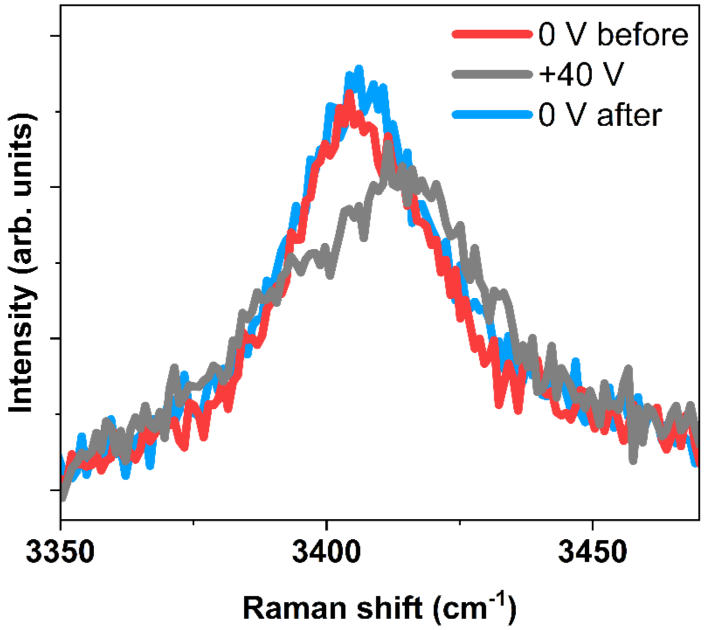

Supplementary Figure 2: Reversibility of O-H$_A$ stretching peak splitting before and after applying external field. Spectra were taken at 300 K on a graphite/hBN/gypsum/hBN/graphite heterostructure. The electric field at 40 V is 0.31 V/nm, taking into consideration the thickness of gypsum film.

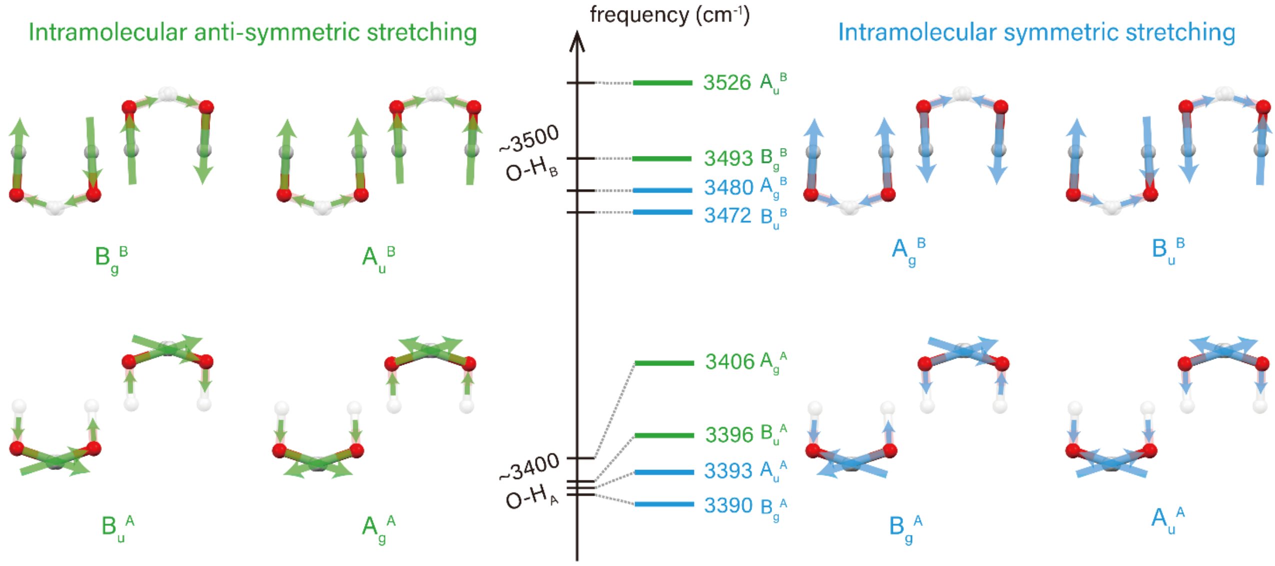

Supplementary Figure 3: Decoupling of the O-H stretching frequencies in crystalline water of gypsum. Vibration levels of symmetric and antisymmetric stretching are sketched by blue and green solid lines. Blue and green arrows represent the vibration amplitudes of symmetric and antisymmetric stretching, respectively. Longer arrows mean larger vibration amplitudes (the amplitudes are exaggerated for clarity). Symmetric and anti-symmetric modes are labelled by A and B, respectively. If the sum of polarizability of the four water molecules changes during vibration, that vibration mode is assigned with a gerade (subscript g) character and is Raman-active. If the sum of dipole moments changes, that vibration mode is assigned with an ungerade (subscript u) character, which is IR-active. Superscripts A and B indicate the mode is from O-H_A or O-H_B vibration.

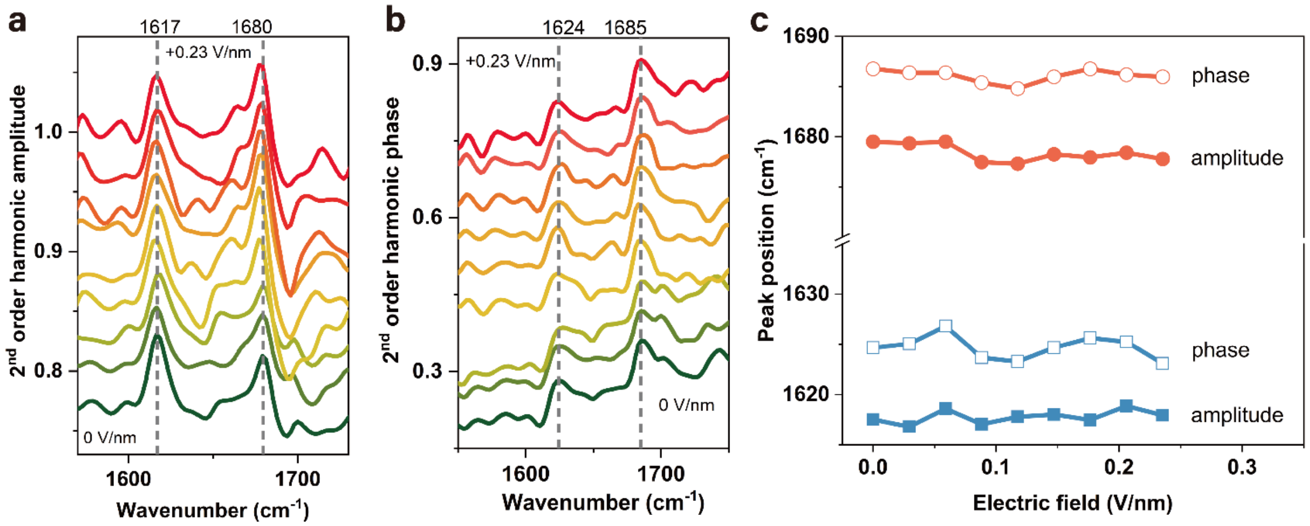

Supplementary Figure 4: Response of water bending mode to external electric field. a-b, nano-FTIR spectra of water bending modes measured using scanning near-field optical microscopy (SNOM). The signal from 2nd harmonic amplitude (**a**) and phase (**b**) are obtained. Two bending vibrations are excited due to the measurement geometry where the incident laser makes an oblique angle to sample stage. **c**, Peak evolution with external electric field along out-of-plane direction. Peak positions extracted from amplitude and phase spectra are plotted as solid and open symbols, respectively. No *E*-field dependence was observed at fields up to 0.23 V/nm.

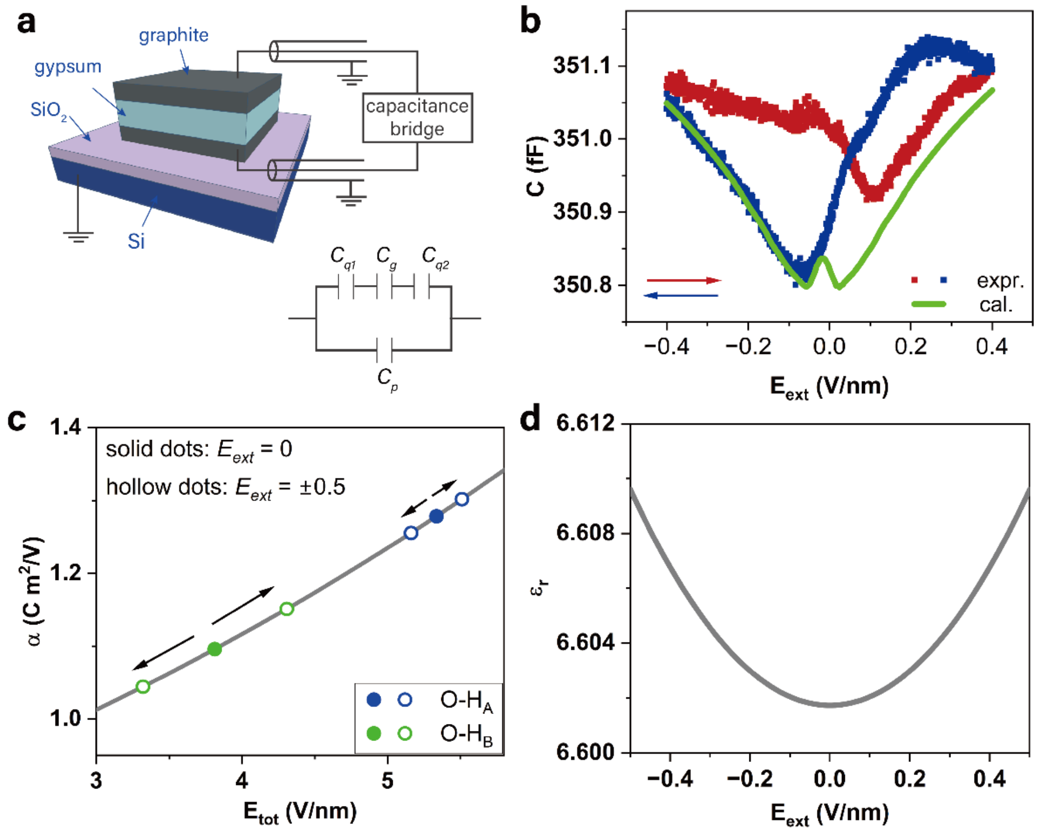

Supplementary Figure 5: Capacitance measurements and dielectric properties of confined water in gypsum. **a**, Schematic of capacitance measurements. During the measurement, the silicon back gate and the shielding of coaxial cables were grounded to minimize parasitic capacitance contributions. The equivalent circuit of the measurement is sketched, with C_g, C_{q1} (C_{q2}) and C_p being capacitance of gypsum, quantum capacitance of top (bottom) graphite electrode and parasitic capacitance of the setup, respectively. **b**, Capacitance C(E_{ext}) of the graphite/gypsum/graphite heterostructure at 300 K. Arrows denote the sweep direction of E_{ext}. Bias voltage applied between top and bottom graphite electrodes was converted to electric field E_{ext} using the thickness of gypsum determined from AFM measurements (50 nm). Green curve is the calculated total capacitance using Eq. 15. **c**, Calculated polarizability (α) along the O-H bond as a function of total electric field using Eq. 14. Here the total field consists of both internal E_{HB} and the applied E_{ext}. Black arrows denote the modulation of α by E_{ext} (E_{ext} = 0, solid dots; E_{ext} = ±0.5 V/nm, hollow dots). **d**, Calculated evolution of ε_r in ultra-confined water in gypsum as a function of E_{ext}.

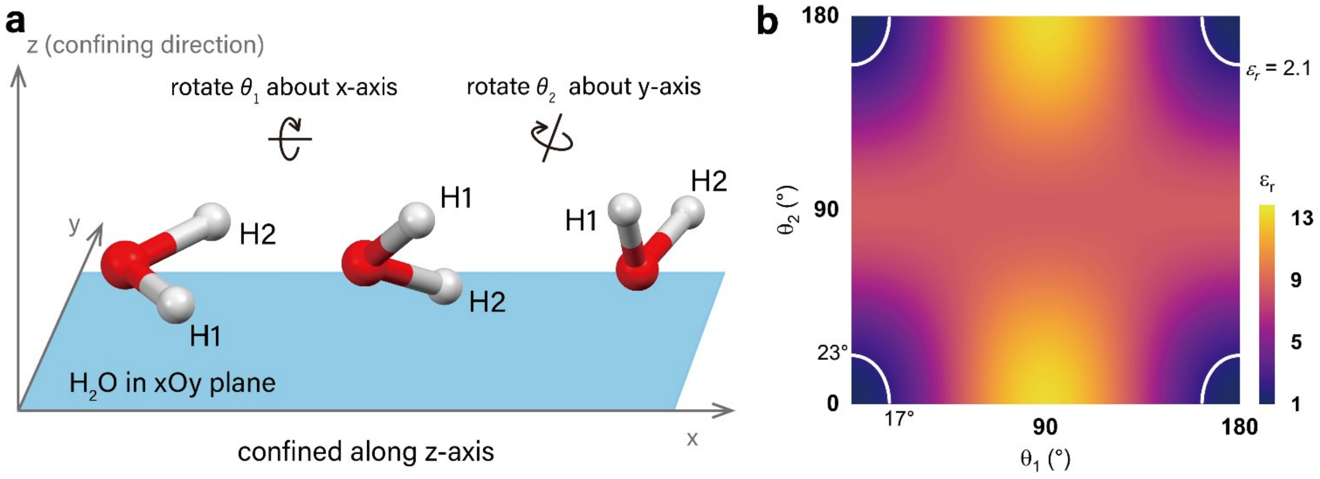

Supplementary Figure 6: Dielectric constant of confined water. a, Schematic of water geometry under confinement. The water molecule is confined along z-direction and has an initial position within xOy plane. The coordinates of O, H1 and H2 are (0,0,0), (cos δ,sin δ,0) and (cos δ,-sin δ,0). δ is the H-O-H angle. To an arbitrary orientation in 3D space, the water molecule first rotates about x-axis by θ_1, followed by a second rotation about y-axis by θ_2**.** Since rotation about z-axis has no effect on its dielectric properties along confining direction, no z-rotation is considered here. **b**, Dependence of ε_r on θ_1 and θ_2. The white lines mark the orientation which gives experimental ε_r = 2.1.[1]

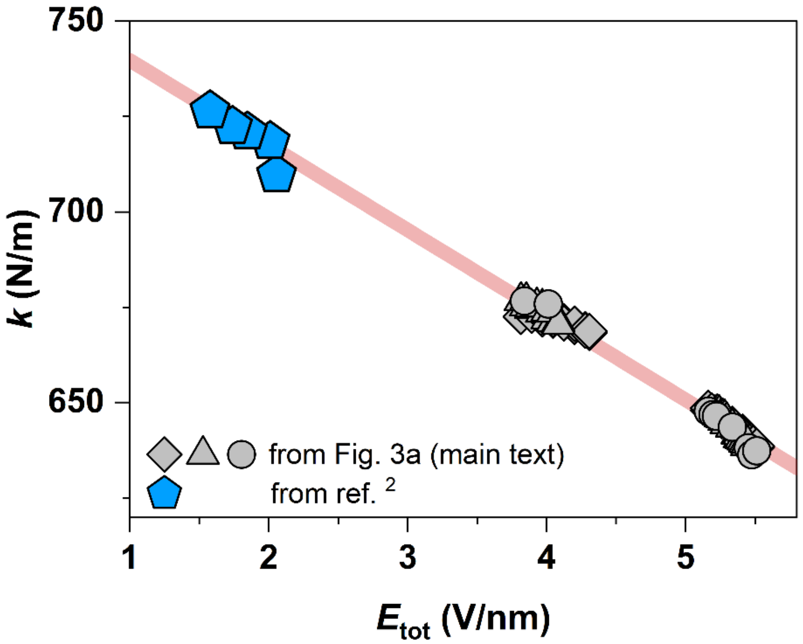

Supplementary Figure 7: Force constant *k* vs E_{tot} along O-H bond. Grey symbols are experimental data of this work, same as in the main text Fig. 3a. Blue pentagons are data of water O-H dipoles at water-graphene interface, taken from ref. [2]. Red line is *k*(*E*) relation given by our model (same as in Fig. 3a in the main text). The size of data symbol and the thickness of fitting line do not represent an error bar.

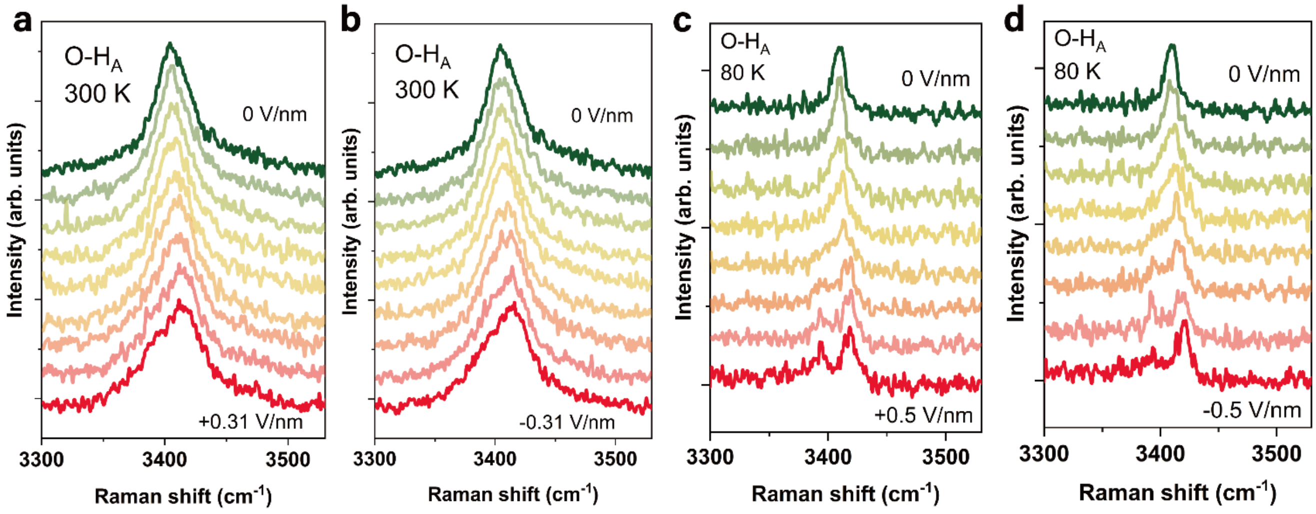

Supplementary Figure 8: Raman spectra of O-H$_A$ splitting under E_{ext}. Peak splitting under room temperature (300 K) (**a-b**) is less pronounced than at 80 K (**c-d**) due to the thermal broadening effect. Such broadening phenomenon was also observed in other temperature-dependent Raman study of gypsum.[3]

Supplementary Table 1: Raman frequency of O-H stretching and neutron diffraction bond length in different confined-water systems used in Fig. 3b in the main text.

	O-H vibration		O-H bond length (thermal-corrected)	
	Raman shift (cm^{-1})	Source	O-H bond length (Å)	Source
**Ice Ih**	3279[a]	Ref. [4]	1.014 ± 0.0011[b]	Ref. [5]
**Ice II**	3194	Ref. [6,7]	1.102 ± 0.021[b]	Ref. [8]
**Ice VI**	3359.5[a]	Ref. [9]	1.010 ± 0.028[b]	Ref. [10]
**Ice VIII**	3427.9	Ref. [11]	1.010 ± 0.009[b]	Ref. [12]
**Ice IX**	3280.7	Ref. [13]	0.983 ± 0.004[b]	Ref. [14]
**Ice XVII**	3106.3	Ref. [15]	1.075 ± 0.007[b]	Ref. [16]
	3231.8		1.049 ± 0.004[b]	
**$CaSO_4 \cdot 2H_2O$**	3406	This work	1.014 ± 0.006[b]	Ref. [17]
	3494		0.995 ± 0.004[b]	
**$Li_2SO_4 \cdot H_2O$**	3439	Ref. [18]	1.004 ± 0.003	Ref. [19]
	3480		0.997 ± 0.002	
**$CuSO_4 \cdot 5H_2O$**	3360	Ref. [20,21]	1.005 ± 0.005	Ref. [22]
	3477		0.951 ± 0.011	
**$Ba(ClO_3)_2 \cdot H_2O$**	3547	Ref. [23]	0.958 ± 0.011	Ref. [24]
**$LiClO_4 \cdot 3H_2O$**	3553	Ref. [25]	0.993 ± 0.004	Ref. [26]
**$BaCl_2 \cdot 2H_2O$**	3303	Ref. [27]	1.077 ± 0.004	Ref. [28]
	3318		1.072 ± 0.003	
	3352		1.0364 ± 0.0030	
	3456		1.0359 ± 0.0030	

[a]Decoupled O-H vibration. [b]Measured from D_2O water.

Supplementary Table 2: Comparison of dipole moments and HB energies between reported values from references and our model estimates.

	Dipole moment (D)		U_{HB} (kcal mol^{-1})		ε_r	
	Ref.	This work	Ref.	This work	Ref.	This work
**Water vapor**	1.855 ± 0.001[29]	1.88 ± 0.04	—	—	—	—
**Liquid water**	2.9 ± 0.6[30]	2.66 ± 0.13	5.26[31]	5.1 ± 0.5	79 (G_k = 2.72-3.70[32])	79 (G_k = 3.15)
**Ice Ih**	3.09 ± 0.03[33]	3.39 ± 0.24*	7.04[34,35]	10.5 ± 1.2	—	—
**2D water (gypsum)**	—	—	—	—	—	6.6 ± 0.7
**$CaSO_4$ wall (gypsum)**	—	—	—	—	2.7 ± 0.02[36] (β-anhydrite)	2.8 ± 0.5
**Ice XI**	3.3[37]	3.39 ± 0.24*	—	—	2.9[38]	3.8 ± 0.5
**Ice II**	—	—	—	—	4.2[39]	5.9 ± 0.8
**Ice IX**	—	—	—	—	4[40]	4.7 ± 0.4
**Water in C_{60}[41]**	—	—	—	1.5 ± 0.2	—	—

* This value is estimated based on the proton-order ice Ih structure, which is effectively ice XI.

1 Fumagalli, L. *et al.* Anomalously low dielectric constant of confined water. *Science* **360**, 1339-1342 (2018).

2 Xu, Y., Ma, Y.-B., Gu, F., Yang, S.-S. & Tian, C.-S. Structure evolution at the gate-tunable suspended graphene–water interface. *Nature* **621**, 506-510 (2023).

3 Chio, C. H., Sharma, S. K. & Muenow, D. W. Micro-Raman studies of gypsum in the temperature range between 9 K and 373 K. *American Mineralogist* **89**, 390-395 (2004).

4 Scherer, J. R. & Snyder, R. G. Raman intensities of single crystal ice I h. *The Journal of Chemical Physics* **67**, 4794-4811 (1977).

5 Kuhs, W. F. & Lehmann, M. S. The structure of the ice Ih by neutron diffraction. *The Journal of Physical Chemistry* **87**, 4312-4313 (1983). https://doi.org:10.1021/j100244a063

6 Taylor, M. & Whalley, E. Raman spectra of ices Ih, Ic, II, III, and V. *The Journal of Chemical Physics* **40**, 1660-1664 (1964).

7 Minceva-Sukarova, B., Sherman, W. & Wilkinson, G. The Raman spectra of ice (Ih, II, III, V, VI and IX) as functions of pressure and temperature. *Journal of Physics C: Solid State Physics* **17**, 5833 (1984).

8 Kamb, B., Hamilton, W. C., LaPlaca, S. J. & Prakash, A. Ordered proton configuration in ice II, from single-crystal neutron diffraction. *The Journal of Chemical Physics* **55**, 1934-1945 (1971).

9 Thoeny, A. V., Parrichini, I. S., Gasser, T. M. & Loerting, T. Raman spectroscopy study of the slow order-order transformation of deuterium atoms: Ice XIX decay and ice XV formation. *J Chem Phys* **156**, 154507 (2022). https://doi.org:10.1063/5.0087592

10 Kuhs, W. F., Finney, J. L., Vettier, C. & Bliss, D. V. Structure and hydrogen ordering in ices VI, VII, and VIII by neutron powder diffraction. *The Journal of Chemical Physics* **81**, 3612-3623 (1984). https://doi.org:10.1063/1.448109

11 Wong, P. & Whalley, E. Raman spectrum of ice VIII. *The Journal of Chemical Physics* **64**, 2359-2366 (1976).

12 Jorgensen, J. D., Beyerlein, R., Watanabe, N. & Worlton, T. Structure of D2O ice VIII from insitu powder neutron diffraction. *The Journal of chemical physics* **81**, 3211-3214 (1984).
13 Bertie, J. E. & Francis, B. F. Raman spectra of the O–H and O–D stretching vibrations of ices II and IX to 25 K at atmospheric pressure. *The Journal of Chemical Physics* **72**, 2213-2221 (1980).
14 La Placa, S. J., Hamilton, W. C., Kamb, B. & Prakash, A. On a nearly proton-ordered structure for ice IX. *The Journal of Chemical Physics* **58**, 567-580 (1973).
15 Del Rosso, L. *et al.* Cubic ice Ic without stacking defects obtained from ice XVII. *Nature Materials* **19**, 663-668 (2020).
16 del Rosso, L. *et al.* Refined structure of metastable ice XVII from neutron diffraction measurements. *The Journal of Physical Chemistry C* **120**, 26955-26959 (2016).
17 Schofield, P. F., Knight, K. S. & Stretton, I. C. Thermal expansion of gypsum investigated by neutron powder diffraction. *American Mineralogist* **81**, 847-851 (1996).
18 Canterford, R. & Ninio, F. Raman study of some water modes in lithium sulphate monohydrate. *Solid State Communications* **15**, 1451-1452 (1974).
19 Lundgren, J. O., Kvick, Å., Karppinen, M., Liminga, R. & Abrahams, S. Neutron diffraction structural study of pyroelectric Li2SO4· H2O at 293, 80, and 20 K. *The Journal of chemical physics* **80**, 423-430 (1984).
20 Berger, J. Infrared and Raman spectra of CuSO4, 5H2O; CuSO4, 5D2O; and CuSeO4, 5H2O. *Journal of Raman Spectroscopy* **5**, 103-114 (1976).
21 Liu, D. & Ullman, F. Raman spectrum of CuSO4· 5H2O single crystal. *Journal of Raman spectroscopy* **22**, 525-528 (1991).
22 Bacon, G. & Titterton, D. Neutron-diffraction studies of CuSO4· 5H2O and CuSO4· 5D2O. *Zeitschrift für Kristallographie-Crystalline Materials* **141**, 330-341 (1975).
23 Bertie, J. E., Heyns, A. M. & Oehler, O. The Infrared and Raman Spectra of Powdered and Single-Crystal Barium Chlorate Monohydrate and-deuterate. *Canadian Journal of Chemistry* **51**, 2275-2289 (1973).
24 Sikka, S., Momin, S., Rajagopal, H. & Chidambaram, R. Neutron-diffraction refinement of the crystal structure of barium chlorate monohydrate Ba (ClO3) 2· H2O. *The Journal of Chemical Physics* **48**, 1883-1889 (1968).
25 Zhang, Y.-H. & Chan, C. K. Observations of water monomers in supersaturated NaClO4, LiClO4, and Mg (ClO4) 2 droplets using Raman spectroscopy. *The Journal of Physical Chemistry A* **107**, 5956-5962 (2003).
26 Lundgren, J.-O., Liminga, R. & Tellgren, R. Neutron diffraction refinement of pyroelectric lithium perchlorate trihydrate. *Acta Crystallographica Section B: Structural Crystallography and Crystal Chemistry* **38**, 15-20 (1982).
27 Lutz, H., Pobitschka, W., Frischemeier, B. & Becker, R.-A. Lattice Vibration Spectra. XX. Infrared and Raman Spectra of BaCl 2• 2H 2 O and BaCl 2• 2D 2 O. *Applied Spectroscopy* **32**, 541-547 (1978).
28 Padmanabhan, V., Busing, W. & Levy, H. A. Barium chloride dihydrate by neutron diffraction. *Acta Crystallographica Section B: Structural Crystallography and Crystal Chemistry* **34**, 2290-2292 (1978).
29 Clough, S. A., Beers, Y., Klein, G. P. & Rothman, L. S. Dipole moment of water from Stark measurements of H2O, HDO, and D2O. *The Journal of Chemical Physics* **59**, 2254-2259 (1973).
30 Badyal, Y. *et al.* Electron distribution in water. *The Journal of Chemical Physics* **112**, 9206-9208 (2000).
31 Lide, D. R. *CRC handbook of chemistry and physics*. Vol. 85 (CRC press, 2004).
32 Yu, H., Hansson, T. & van Gunsteren, W. F. Development of a simple, self-consistent polarizable model for liquid water. *The Journal of chemical physics* **118**, 221-234 (2003).
33 Batista, E. R., Xantheas, S. S. & Jónsson, H. Molecular multipole moments of water molecules in ice Ih. *The Journal of chemical physics* **109**, 4546-4551 (1998).
34 Whalley, E. Energies of the phases of ice at zero temperature and pressure. *The Journal of chemical physics* **81**, 4087-4092 (1984).

35 Nanayakkara, S., Tao, Y. & Kraka, E. Capturing individual hydrogen bond strengths in ices via periodic local vibrational mode theory: Beyond the lattice energy picture. *Journal of Chemical Theory and Computation* **18**, 562-579 (2021).
36 López-Buendía, A. M., García-Baños, B., Urquiola, M. M., Catalá-Civera, J. M. & Penaranda-Foix, F. L. Evidence of a new phase in gypsum–anhydrite transformations under microwave heating by in situ dielectric analysis and Raman spectroscopy. *Physical Chemistry Chemical Physics* **22**, 27713-27723 (2020).
37 Ishii, F., Terada, K. & Miura, S. First-principles study of spontaneous polarisation and water dipole moment in ferroelectric ice XI. *Molecular Simulation* **38**, 369-372 (2012).
38 Shimizu, N. & Muramoto, Y. Natural materials adopted for electrical and electronic materials. *IEEJ Transactions on Fundamentals and Materials* **127**, 429-432 (2007).
39 Wilson, G., Chan, R., Davidson, D. & Whalley, E. Dielectric properties of ices II, III, V, and VI. *The journal of chemical physics* **43**, 2384-2391 (1965).
40 Whalley, E., Heath, J. & Davidson, D. Ice IX: an antiferroelectric phase related to ice III. *The Journal of Chemical Physics* **48**, 2362-2370 (1968).
41 Shugai, A. *et al.* Infrared spectroscopy of an endohedral water in fullerene. *J Chem Phys* **154**, 124311 (2021). https://doi.org:10.1063/5.0047350
